# Navigating the labyrinth of peritoneal and extraperitoneal anatomy: abdominal spread made easy with a case based review

**DOI:** 10.1007/s00261-024-04429-y

**Published:** 2024-06-21

**Authors:** Praveen Polamraju, Michael Oliphant, Swetha Aribindi, Janardhana Ponnatapura

**Affiliations:** Department of Diagnostic Radiology, Wake Forest University School of Medicine, 1, Medical Center Blvd, Winston Salem, NC 27157 USA

**Keywords:** Peritoneum, Anatomy, Mesentery, Ligaments

## Abstract

Essential to understanding disease spread in abdomen is to separate the peritoneum from the extraperitoneum. These areas have distinct anatomy with well-define separate pathways. The peritoneum is comprised of connected recesses that are potential spaces, normally not imaged except when containing excess fluid or air. Peritoneal recesses are formed by the opposing peritoneal surfaces and subdivided by the attachments of the ligaments and mesenteries to the parietal peritoneum. Disease flows within the recesses by changes in abdominal pressure. This forms a distinct spread pattern. The extraperitoneum is traditionally stratified by the renal fascia into the anterior and posterior pararenal spaces and the perirenal space. The fascia contains and directs spread from the contained organs with the compartments. Each space has a unique spread pattern defined by the containing fascia. The extraperitoneum is connected to the mesenteries and ligaments forming the subperitoneal space. This space interconnects the extraperitoneum with the mesenteries allowing for the normal continuum of blood vessels, lymphatics, and nerves but also forms the pathways for bidirectional spread of disease.

## Learning objectives

- Understand what constitutes the peritoneal recesses and extraperitoneal spaces
- Understand their distinct pathways of disease spread
- Diagnostic importance of recognizing the different disease spread patterns in the peritoneal recesses compared to the extraperitoneal spaces
- Understand the concept of the subperitoneal spread interconnecting the extraperitoneum to the mesentery for bidirectional spread of disease

## Introduction

Understanding disease spread in the abdomen requires a recognition of the differences between the peritoneum and extraperitoneum [1–4]. Peritoneal spread of disease has a distinct pattern dictated by its anatomy and flow pattern. The extraperitoneal space is defined as the space between the peritoneum and transversalis fascia [2, 5]. Posteriorly, the extraperitoneum is stratified by the anterior and posterior renal fascia into the pararenal space, perirenal space, and posterior pararenal space [2]. Disease spread within these spaces is dictated by fascial planes [6–13]. The subperitoneal space is an extension of the extraperitoneal space into the suspended abdominal viscera via their corresponding mesenteries [1–5, 14–16]. Subperitoneal spread of disease occurs along the avenues formed by the vessels connecting the intraperitoneal spaces and the mesenteries.

### Peritoneum

**Fig. 1:**
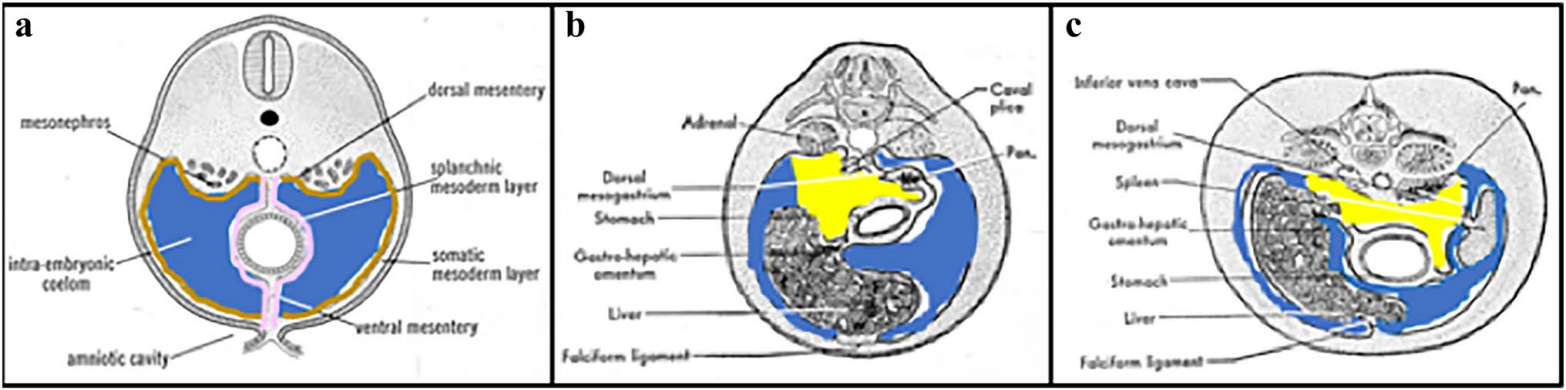
Illustrations of early abdominal embryological development demonstrate the layers of peritoneum and cavities forming over time from (**a**) through (**c**). Initially, the ventral recesses form in (**a**). Over time, the dorsal recesses and lesser sac form after organal rotation and mesentery elongation. A brown outline in **a** depicts the parietal layer and a light pink outline in **a** depicts the visceral layer. Blue shading reflects the greater sac and yellow shading show the lesser sac developing in (**b**) and (**c**). Images **a**–**c** reproduced with minor editing from “Meyers, M. A., Charnsangavej, C., Oliphant, M. (2011). *Meyers' dynamic radiology of the abdomen: normal and pathologic anatomy*. New York, NY: Springer” with permission from Springer

The peritoneal cavity is the potential space containing the suspended abdominal viscera and their mesenteries [2, 5, 16–18]. It is formed from the primitive coelom when the lateral mesoderm splits into the somatic and splanchnic layers [2, 18–20]. The somatic mesoderm covered by parietal peritoneum lines the coelomic wall. The splanchnic mesoderm lined by visceral peritoneum covers the suspended viscera and mesenteries (Fig. 1).

**Fig. 2:**
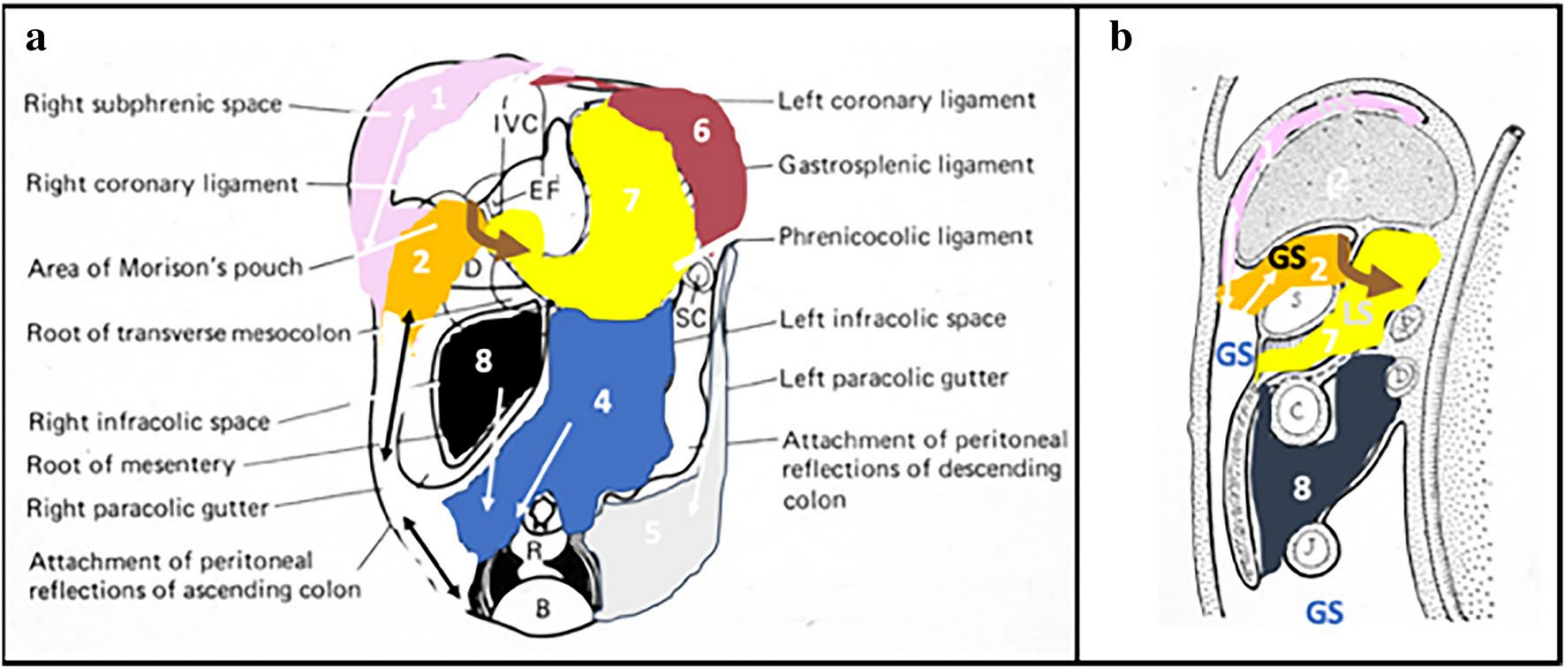
Schematic illustrating **a** coronal and **b** sagittal views depicting peritoneal anatomy and direction of flow in the peritoneal recesses. Anatomy includes the right subphrenic recess (1, pink), right subhepatic recess (2, orange), right paracolic gutter (3, black), left inframesocolic recess (4, blue), left paracolic gutter (5, light gray), right inframesocolic recess (8, dark gray), and left subphrenic recess (6, dark magenta). Ligaments depicted include the phrenicocolic ligament (black line) and falciform ligament (black dashed line). The foramen of Winslow (brown arrow) provides the only passage between the greater and lesser sac (7, yellow). Abbreviations include B-bladder, C-colon, D-duodenum, GS-greater sac, IVC-inferior vena cava, J-jejunum, L-liver, LS-lesser sac, P-pancreas, R-rectum, and S-stomach. Images **a–b** reproduced with minor editing from “Meyers, M. A., Charnsangavej, C., Oliphant, M. (2011). *Meyers' dynamic radiology of the abdomen: normal and pathologic anatomy*. New York, NY: Springer” with permission from Springer

**Fig. 3:**
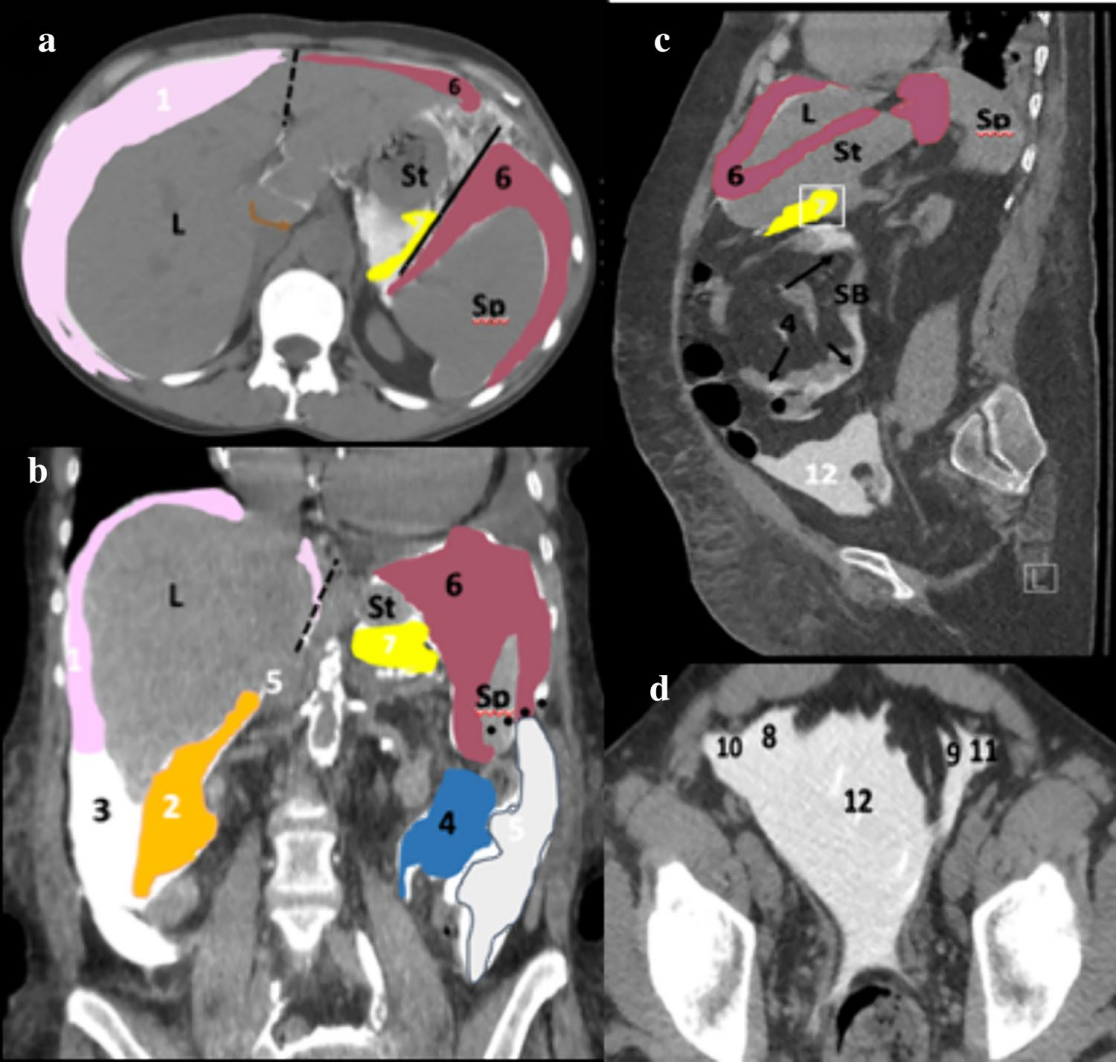
Cases depicting the peritoneal spaces in patients while evaluating for a peritoneal dialysis leak. Axial CT section (**a**), coronal CT section (**b**), and sagittal CT section of the abdomen show contrast within peritoneal recesses. Important peritoneal anatomy includes the right subphrenic recess (1, pink), right subhepatic recess (2, orange), right paracolic gutter (3, white), left inframesocolic recess (4, blue), left paracolic gutter (5, light gray), and left subphrenic recess (6, dark magenta). Ligaments depicted include the phrenicocolic ligament (black dotted line), falciform ligament (black dashed line), and gastrosplenic ligament (black solid line). The foramen of Winslow (brown arrows) provides the only passage between the greater and lesser sac (7, yellow). Inferiorly, pelvic contrast opacifies the right and left medial (8 and 9 respectively) and lateral (10 and 11 respectively) inguinal recesses, as well as the supravesical recess (12) in (**c**) and (**d**). Abbreviations include L-liver, Sp-spleen, SB-small bowel, and St-stomach

**Fig. 4:**
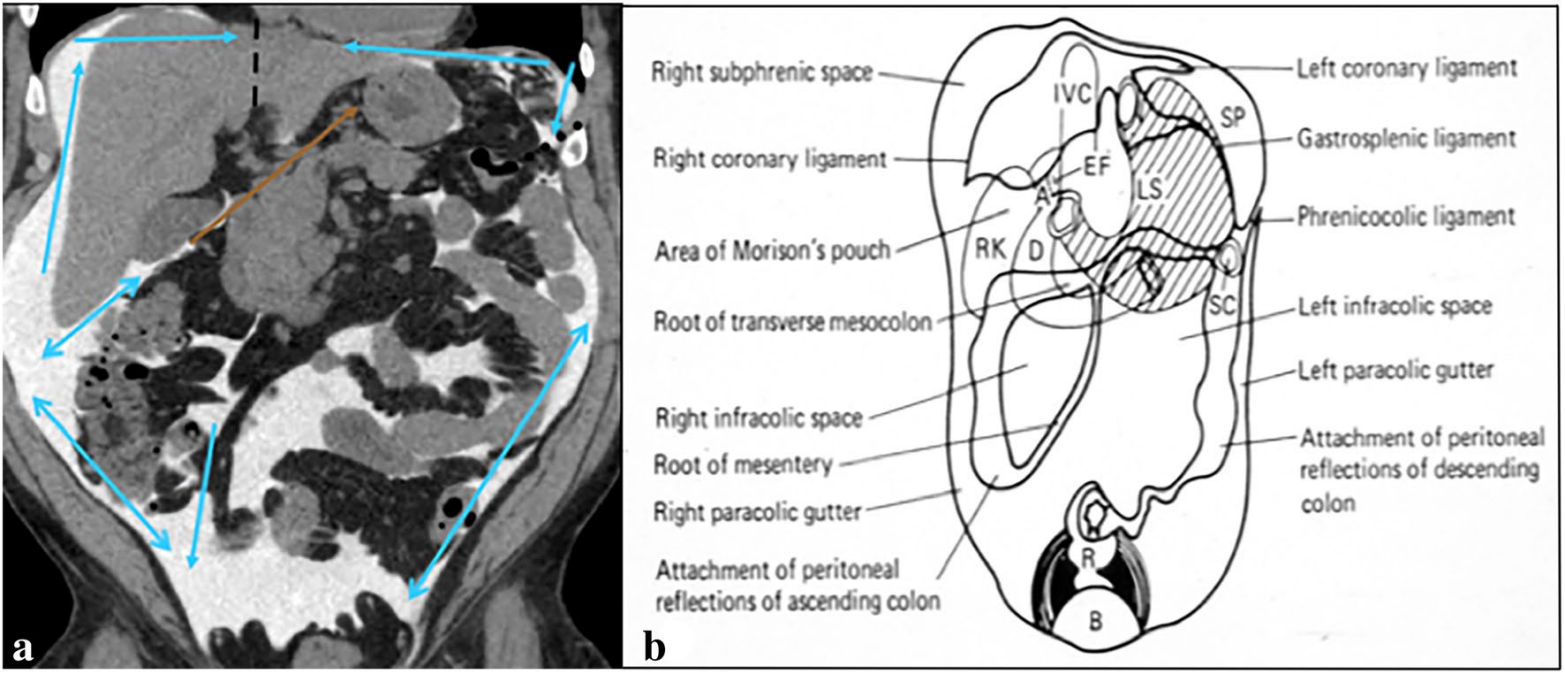
Case continued depicting the peritoneal recesses in a patient evaluating for peritoneal dialysis leak in (**a**). Light blue arrows depict the direction of flow in the peritoneal recesses. A brown arrow approximates the course between the right subhepatic recess into the lesser sac via the foramen of Winslow. Coronal schematic (**b**) providing a correlate depicting flow in the peritoneal recesses. Image **b** reproduced from “Meyers, M. A., Charnsangavej, C., Oliphant, M. (2011). *Meyers' dynamic radiology of the abdomen: normal and pathologic anatomy*. New York, NY: Springer” with permission from Springer

The transverse mesocolon divides the peritoneal cavity into the supramesocolic and inframesocolic portions [2, 19]. The supramesocolic recesses are divided ventrally by the falciform ligament. The right subphrenic space continues lateral to the liver and inferiorly to the subhepatic spaces (Figs. 2, 3). The subhepatic space continues posteromedial to the liver into the lesser sac via the epiploic foramen (Figs. 2, 3 and 4).

The lesser sac is a peritoneal recess formed by the elongation and rotation of the dorsal mesogastrium [2, 5, 19] (Figs. 2,3). It lies posterior to the stomach and anterior to the transverse colon. The lesser sac is bordered by the stomach, duodenum, pancreas, spleen, as well as the gastrohepatic, hepatoduodenal, and gastrosplenic ligaments.

**Fig. 5:**
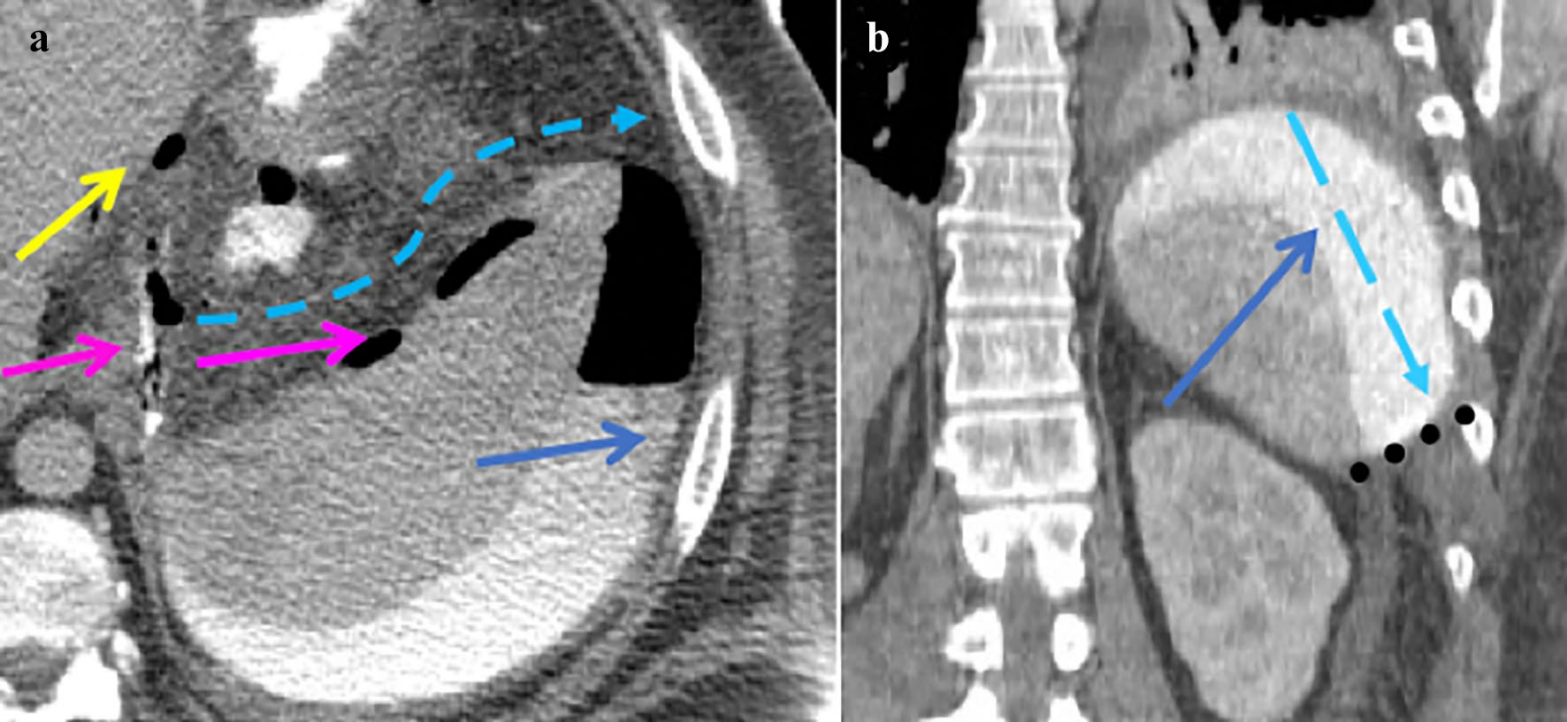
Case illustrating the left subphrenic space in a 36-year-old patient with history of gastric bypass and recent repair of gastro-gastric fistula presenting with left upper quadrant pain. **a** Axial CT and **b** coronal CT sections showing extraluminal enteric contrast and gas (yellow arrows) along the margin of the suture site in the left gastrohepatic recess (subphrenic recess) extending further into the left gastrosplenic recess (subphrenic recess) marked by a pink arrow. Expected flow of contrast depicted by dashed light blue arrows with inferior extent limited by the phrenicocolic ligament (dotted line)

**Fig. 6:**
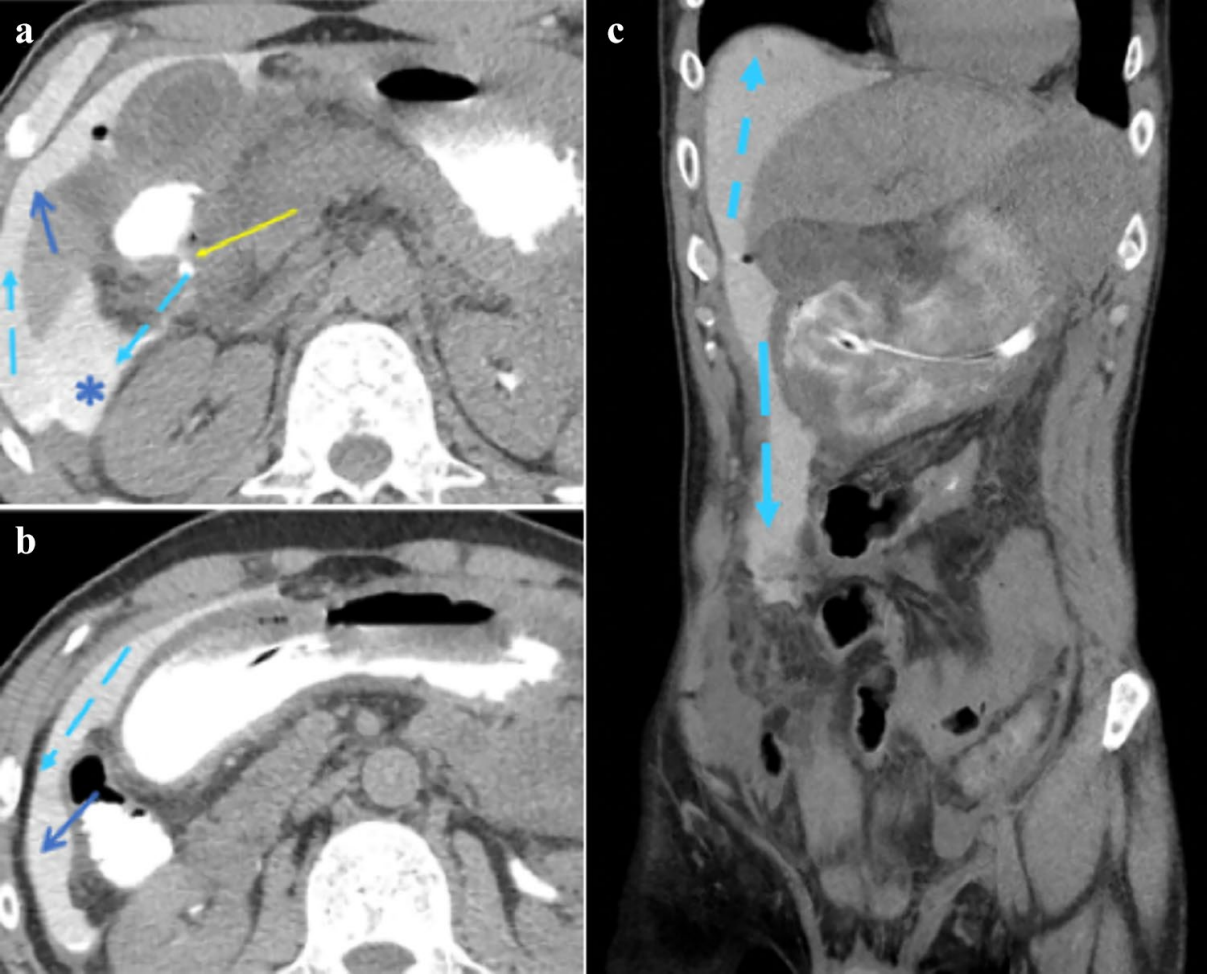
Case illustrating the right subphrenic recess and paracolic gutter in a 54-year-old patient with history of peptic ulcer disease complaining of coffee ground emesis and severe pain. Axial CT section at **a** celiac artery level and **b** superior mesenteric artery level, as well as **c** coronal CT demonstrate extraluminal contrast and gas arising from a site at the second portion of the duodenum (yellow arrow), compatible with a perforated duodenal ulcer. Extraluminal contrast also extends into Morison’s pouch (posterior right subhepatic recess) (blue star), right subphrenic recess (blue arrow in image **a**), and inferiorly into the right paracolic gutter (blue arrow in image **b**). Dashed light blue arrows depict the expected flow of pathology from the site of perforation with superior extent limited by the phrenicocolic ligament (dotted line)

The gastrohepatic and gastrosplenic recesses subdivide the left subphrenic space [5] (Fig. 5). The splenorenal recess is the posterior left supramesocolic recess formed by the splenorenal ligament [5]. The left supramesocolic recesses are separated from the right supramesocolic recesses by the falciform ligament ventrally, lesser sac medially, and inframesocolic recesses laterally the phrenicocolic ligament (Figs. 2, 3, 4, 5 and 6).

**Fig. 7:**
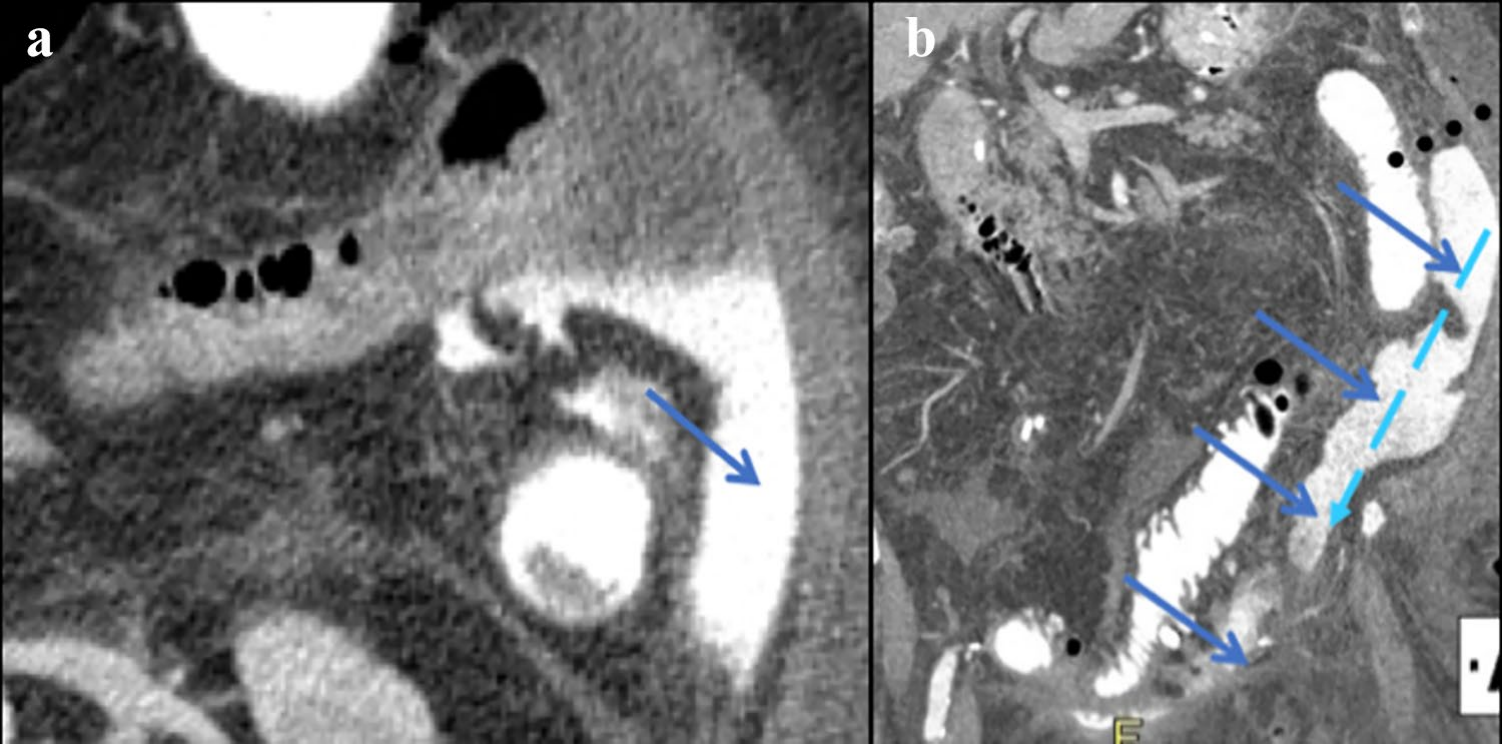
Case illustrating the left paracolic gutter in an 83-year-old patient s/p radical cystectomy and ileal-ileal anastomoses with abdominal pain. **a** Axial and **b** coronal CT sections demonstrates a jejunal perforation with extraluminal contrast, fluid, and gas in the left paracolic gutter (solid blue arrows) in a patient status post radical cystectomy and ileal-ileal anastomosis who presented with abdominal pain. Extraluminal contrast communicates with the peritoneum in the pelvis with flow of contrast depicted by dashed light blue arrows

The right supramesocolic recesses continue to the right inframesocolic recesses by the right paracolic gutter (recess) (Figs. 2,3,6) [2, 5]. This recess is in continuity with the recesses between the small intestine and its mesentery (Fig. 7). The right paracolic gutter continues inferiorly to the pelvic peritoneal recesses. Ventrally, these recesses are divided by the urachus (medial umbilical ligament), the obliterated umbilical arteries (median umbilical ligament, and inferior epigastric vessels (lateral umbilical ligament) into the left and right inguinal recess and supravesical recess [2] (Fig. 3). Dorsally, these recesses are divided in the male by the urinary bladder and rectum to the rectovesical recess and in the female by the rectum, uterus, and urinary bladder to the rectovesical recess (Douglas pouch, cul-de-sac) and the uterovesical recess. The pelvic recesses continue lateral to the paravesical recesses and to the right and left paracolic gutters. Prior literature further details the pelvic peritoneal recesses [2, 5].

**Fig. 8:**
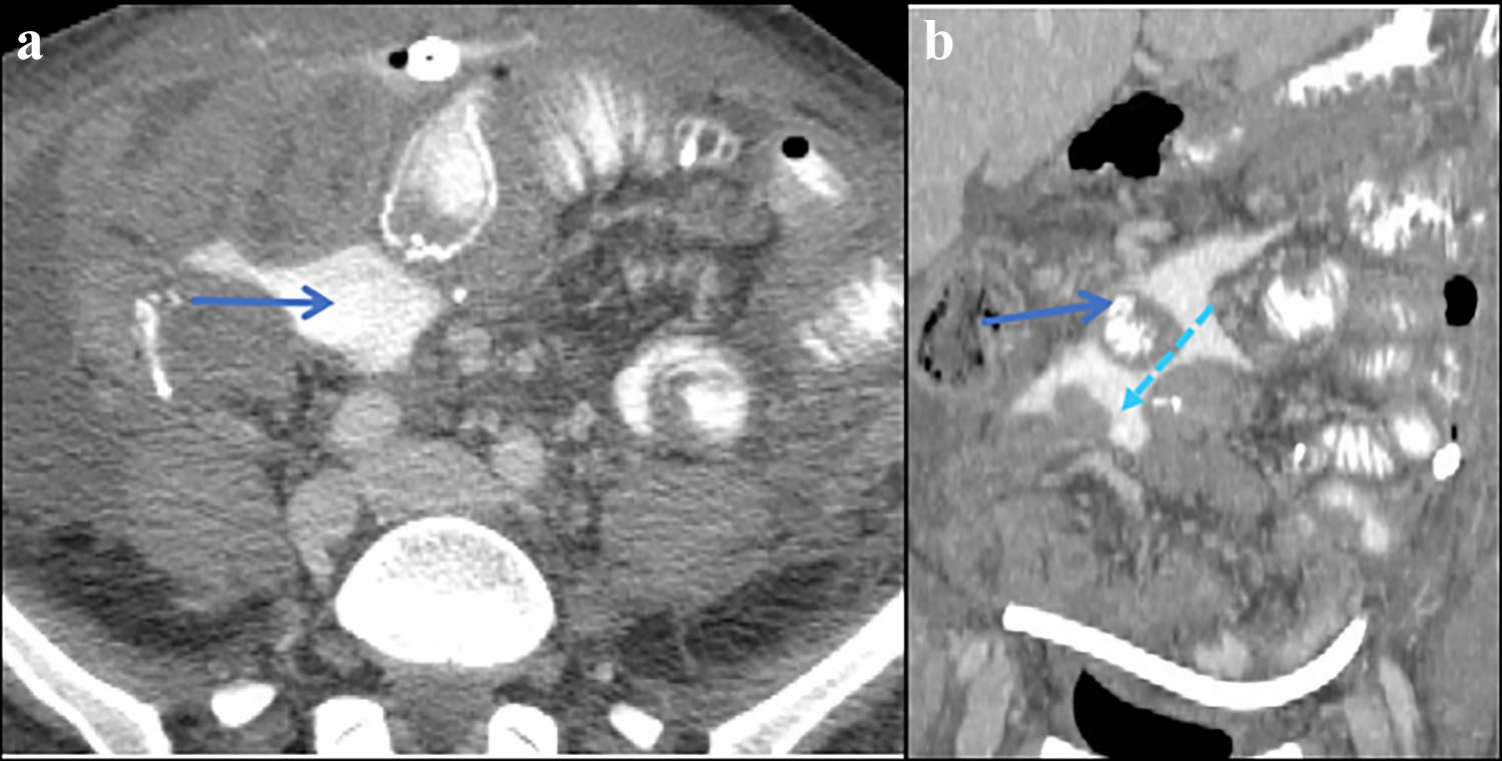
Case illustrating the right inframesocolic recess in a 44-year-old patient with history of appendiceal carcinamatosis, right hemicolectomy, and omentectomy with small bowel resection 1 day ago. **a** Axial and **b** coronal CT sections show extraluminal contrast (blue arrows) in the inframesocolic recess (interloop fluid). Findings are compatible with perforated small bowel. Expected flow of contrast depicted by dashed light blue arrows

**Fig. 9:**
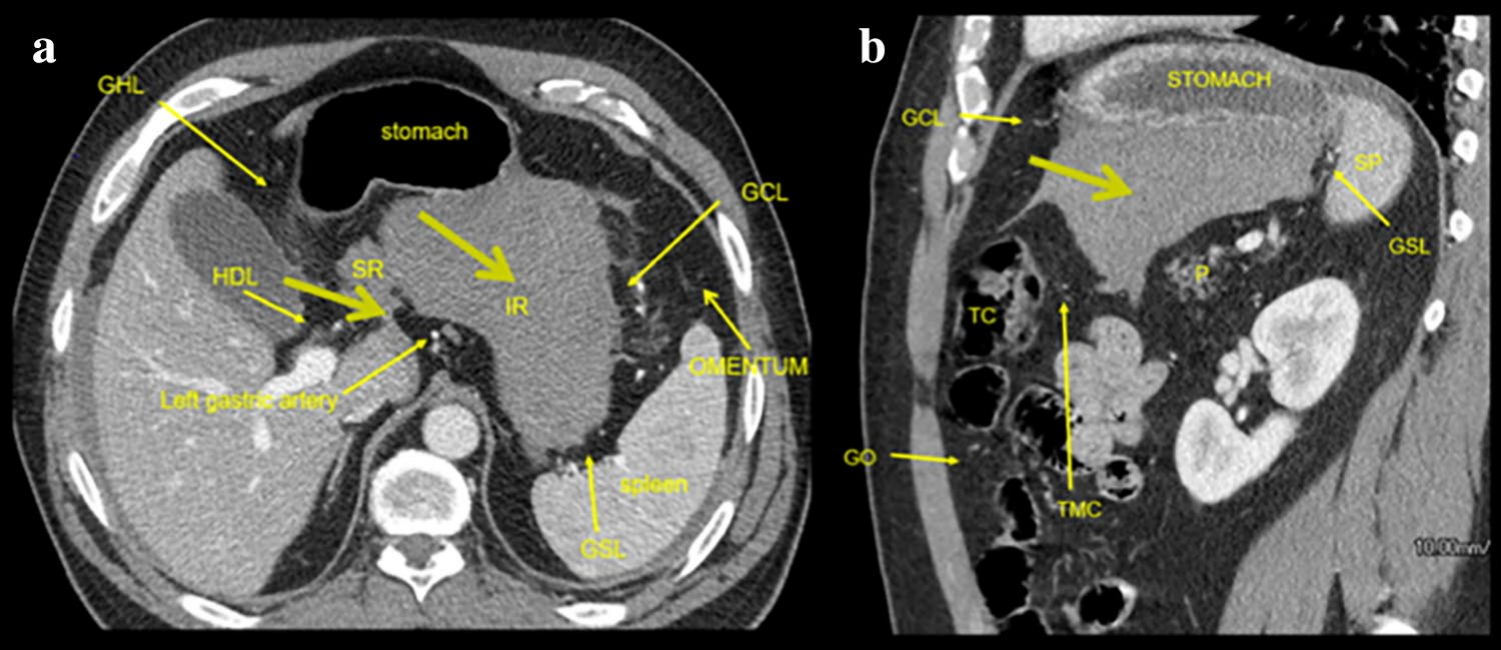
Case illustrating the lesser sac in a 48-year-old male while cutting trees a large branch struck his left flank with bleeding from the splenic artery. **a** Axial CT section and **b** coronal CT section shows a hematoma in the lesser sac (thick olive arrows). Surgery revealed bleeding from a splenic artery branch. Hematoma courses along the superior (SR) and inferior recesses (IR). Ligaments bounding the lesser sac include the gastrocolic ligament (GCL), gastrosplenic ligament (GSL), transverse mesocolon (TMC), and foramen of Winslow. Incidental note of imaged gastrohepatic (GHL) and hepatoduodenal ligament (HDL)

Gravity predominately determines flow within these recesses with contribution from changes in intra-abdominal pressure and gastrointestinal peristalsis [2, 17, 19–21] (Figs. 2, 3). Note that fluid flow is unidirectional from interloop fluid in the inframesocolic recesses (Figs. 2, 3, 8), sequestered in the left subphrenic and posterior supramesocolic recesses (Figs. 2, 3, 5), and in the lesser sac (Figs. 3, 9).

**Fig. 10:**
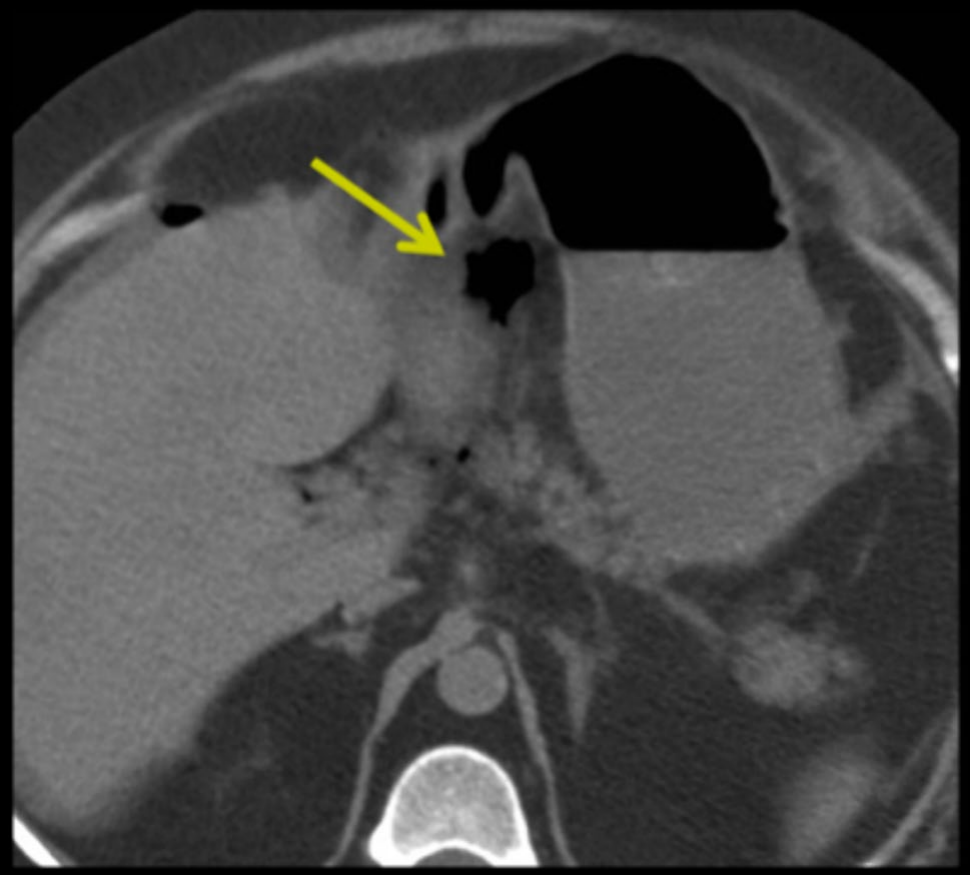
Case illustrating the lesser sac in a 65-year-old patient with severe abdominal pain. Axial CT section shows pneumoperitoneum predominantly in the lesser sac (yellow arrow). At surgery, the patient was found to have a proximal duodenal perforation

The anatomy of the peritoneal recesses and their flow pattern provides a distinctive spread pattern for the peritoneal disease. Examples include tumor penetrating an organ’s peritoneal lining (transperitoneal spread) entering the peritoneal cavity and spread within the recesses to seed the parietal peritoneum and the visceral peritoneum. Fluid and gas from a perforated viscus may spread diffusely through interconnection of many of the peritoneal recesses (Fig. 6). Ligamentous boundaries may limit spread. For instance, the phrenicocolic ligament limits communication between the left inframesolic and supramesocolic recesses (Figs. 5 and 8). Gas or fluid in the lesser sac localizes disease from the surrounding organs (Figs. 9, 10). Interloop fluid localizes pathology to the small intestine or mesentery (Fig. 8). Pathology from the stomach can localize to the left subphrenic space (Fig. 5).

## Extraperitoneum

**Fig. 11:**
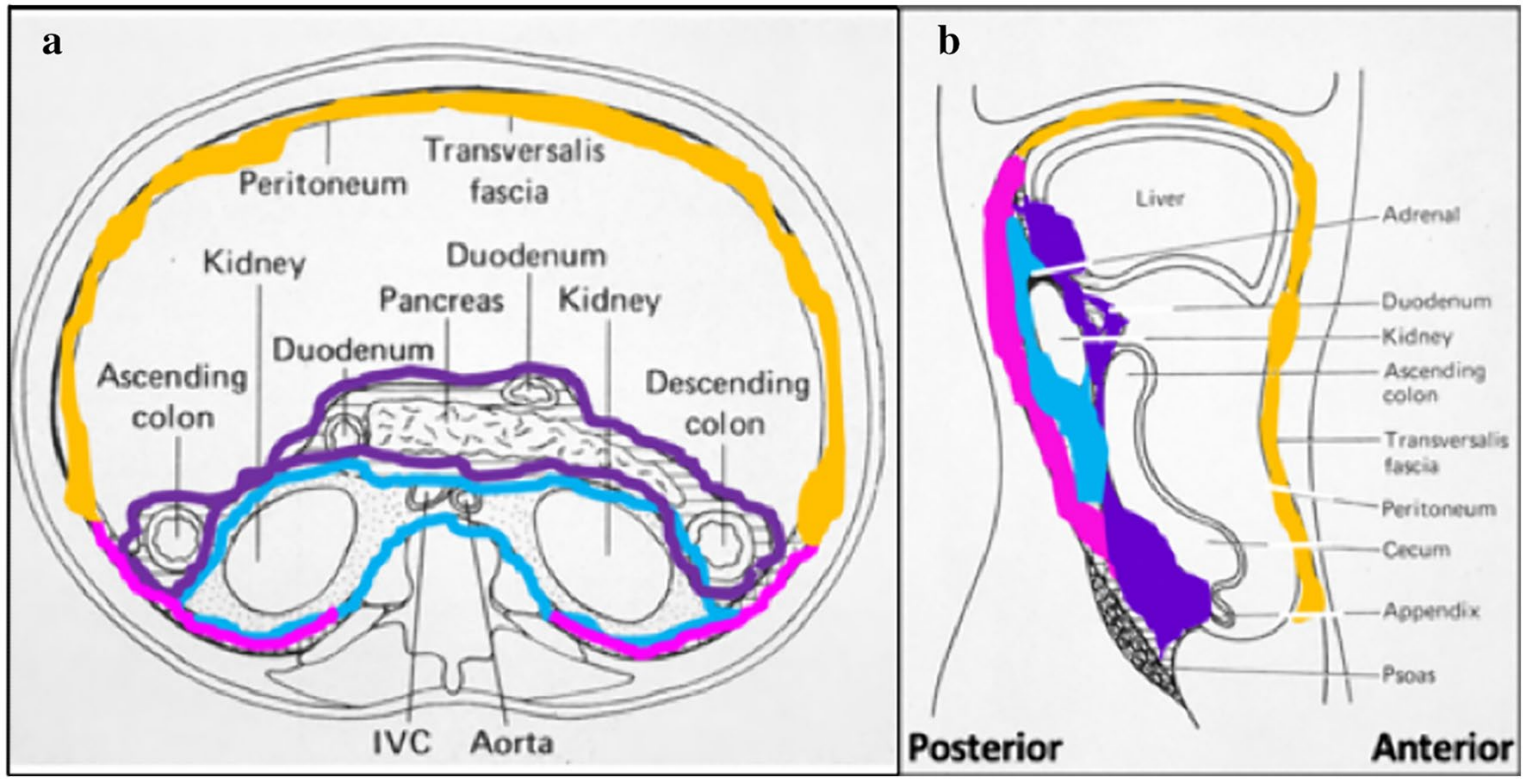
Illustrations depicting an **a** axial view and **b** sagittal view of the abdomen with the traditional retroperitoneal spaces, specifically the anterior pararenal space (dark purple outline/shading), perirenal space (blue outline/shading), and posterior pararenal space (pink outline/shading). The remainder of the extraperitoneal space between the transversalis fascia and peritoneum is shaded in orange. Images **a**, **b** reproduced with minor editing from “Meyers, M. A., Charnsangavej, C., Oliphant, M. (2011). *Meyers' dynamic radiology of the abdomen: normal and pathologic anatomy*. New York, NY: Springer” with permission from Springer

**Fig. 12:**
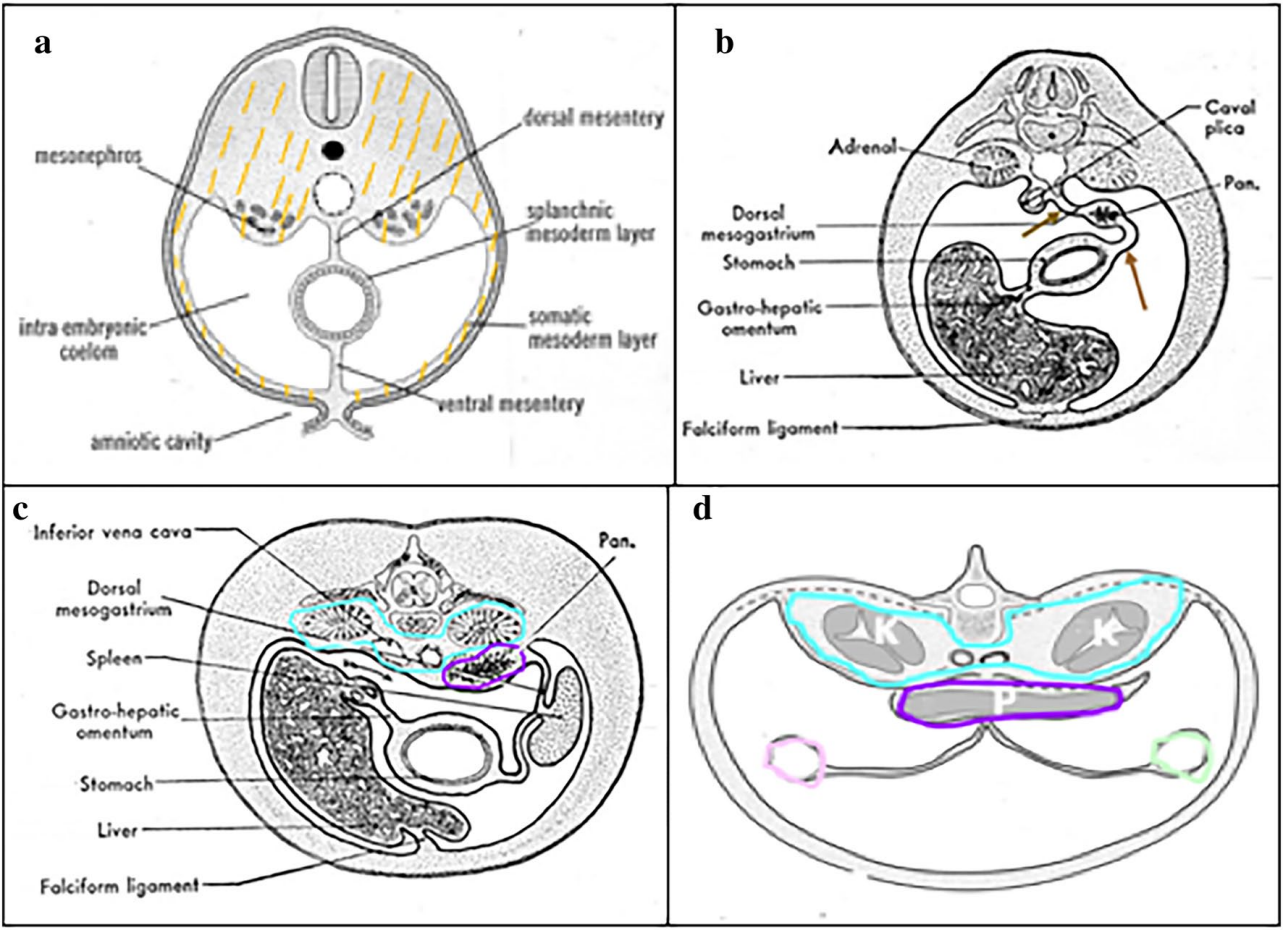
Illustration of early abdominal embryological development demonstrating the primary extraperitoneum (pink outline) outside parietal peritoneum (outlined in Fig. 1) sequentially over time from (**a**) through (**d**). Orange outlines the extraperitoneum in (**a**). The pancreas, spleen, and duodenum form in the dorsal mesogastrium (brown arrows in **b**). The duodenum, pancreas, and respective mesenteries fuse (purple outline in **c** and **d**) onto the extraperitoneum (light blue outline) to form the pancreaticoduodenal compartment. After fusion of the pancreaticoduodenal compartment, the ascending and descending colon form in the dorsal mesentery and fuse onto the anterior pararenal space to form the right (pink) and left (light green) colonic compartments in (**d**). Images **a**–**d** reproduced with minor editing from “Meyers, M. A., Charnsangavej, C., Oliphant, M. (2011). *Meyers' dynamic radiology of the abdomen: normal and pathologic anatomy*. New York, NY: Springer” with permission from Springer

The extraperitoneum is an actual space. It is lies between the peritoneum and transversalis fascia [2, 6, 7] (Fig. 11). Posteriorly, the renal fascia stratifies the extraperitoneum into the anterior and posterior pararenal spaces, as well as the perirenal space containing the kidneys and adrenal glands [2] (Fig. 11). A secondary extraperitoneum forms from fusion of viscera and mesenteries originally developed in the dorsal mesenteries [2, 22, 23] (Fig. 12). The duodenum and pancreas form in the dorsal mesogastrium and fuse onto the anterior pararenal space as the pancreaticoduodenal compartment. The ascending and descending colon form in the dorsal mesentery and fuse onto the anterior pararenal space as the right and left colonic compartments [2, 22, 23] (Fig. 12).

**Fig. 13:**
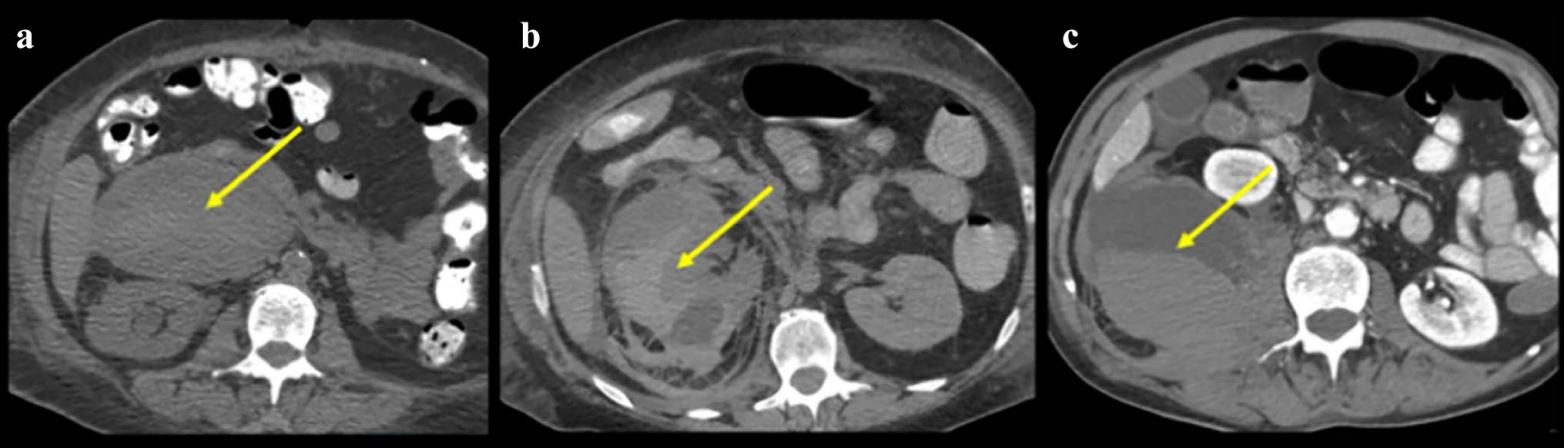
Cases depicting the **a** anterior pararenal space, **b** perirenal space, and **c** posterior pararenal space by collections and/or hematomas on axial CT sections. Pathology preferentially courses inferolaterally in the anterior pararenal space, dorsomedially to the lower pole in the perirenal space, and inferolaterally in the posterior pararenal space

**Fig. 14:**
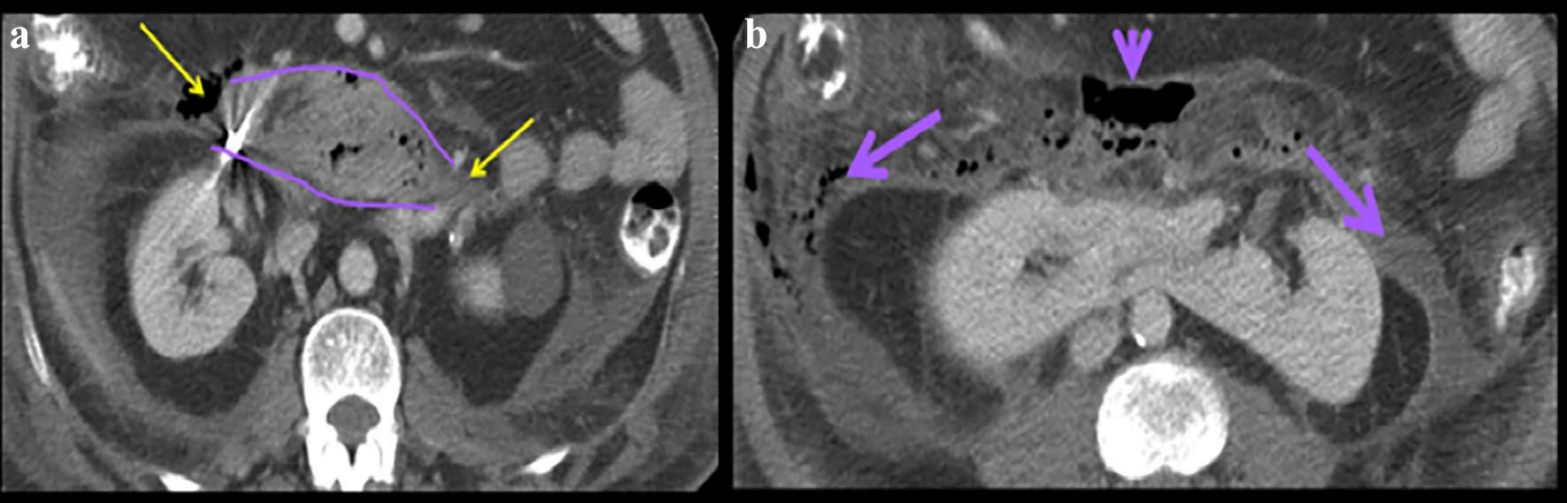
Case illustrating the pancreaticoduodenal compartment in a 47-year-old patient who fell on a lawnmower. Axial CT sections at (**a**) the level of mid pole of the kidney and at (**b**) the level of lower pole of kidney show extraluminal gas and fluid {yellow arrows in image (**a**) and light purple arrows in image (**b**)} in the pancreaticoduodenal compartment of the anterior pararenal space {outlined with light purple lines in image (**a**)} along the course of the duodenum. Extraluminal gas and fluid courses inferolaterally {light arrows in image (**b**)}. The gas and history of trauma allowed for a preoperative diagnosis of duodenal perforation. There is an incidental note of a horseshoe kidney

**Fig. 15:**
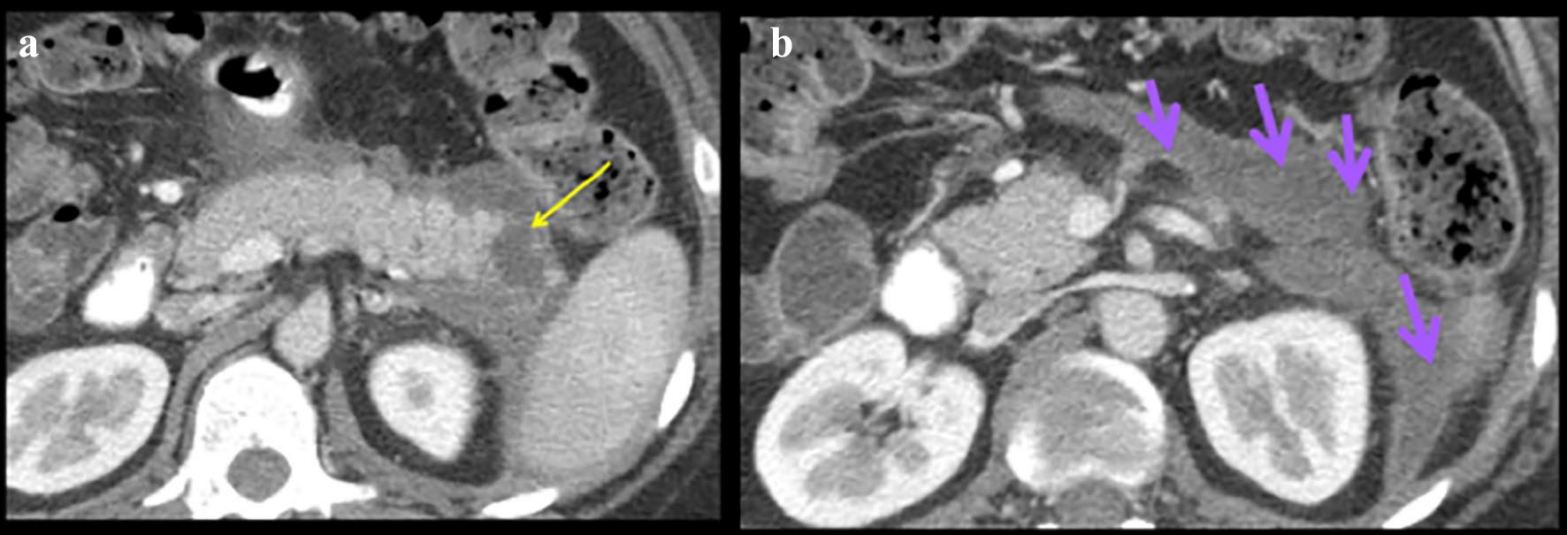
Case illustrating the pancreaticoduodenal compartment in a 56-year-old patient with history of alcohol abuse presenting with abdominal pain. Axial CT sections at the **a** level of body of the pancreas and **b** at the level of uncinate process of pancreas show acute pancreatitis with possible necrosis in the pancreatic tail (yellow arrow in image **a**). A fluid collection in the pancreaticoduodenal compartment of the anterior pararenal space courses inferolaterally (light purple arrows in image **b**). In this case, pathology localizes to the pancreas given acute pancreatitis and peripancreatic collections

**Fig. 16:**
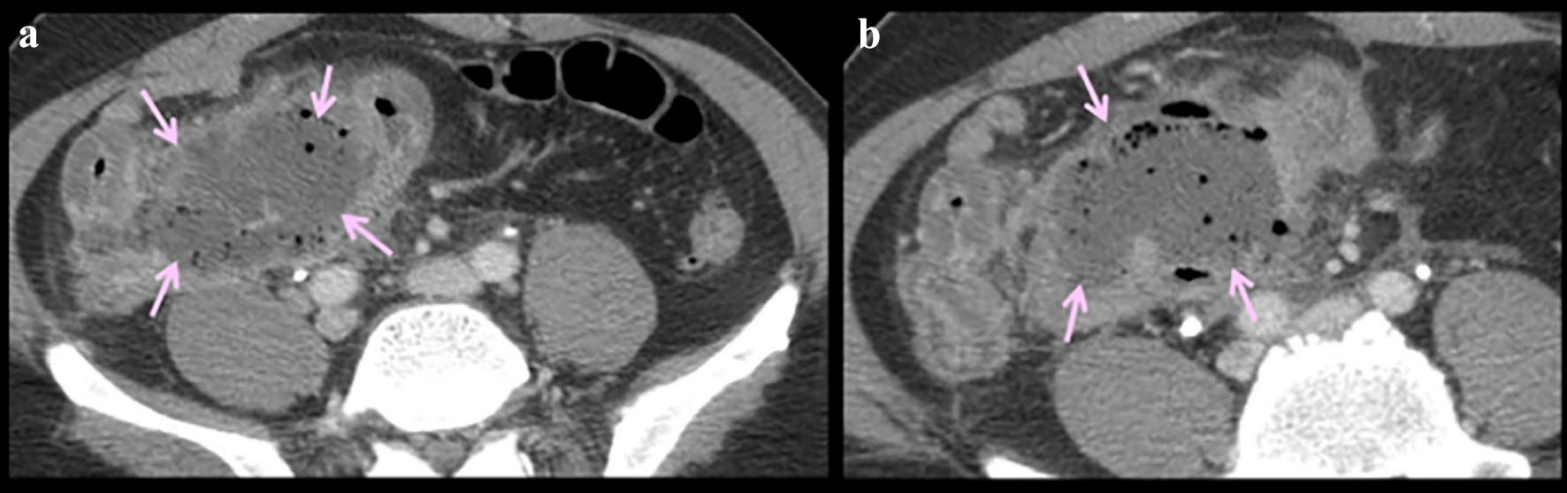
Case illustrating the right colonic compartment in a 27-year-old patient with 4 days of abdominal pain. Fluid and gas collection contained in the anterior pararenal space along the right colonic compartment of the anterior pararenal space (light pink arrows). There is a diagnosis of contained perforation from either the appendix, cecum, or ascending colon. Workup ruled out tumor, diverticular disease, and foreign body. At surgery, this was diagnosed as probable perforated appendicitis

**Fig. 17:**
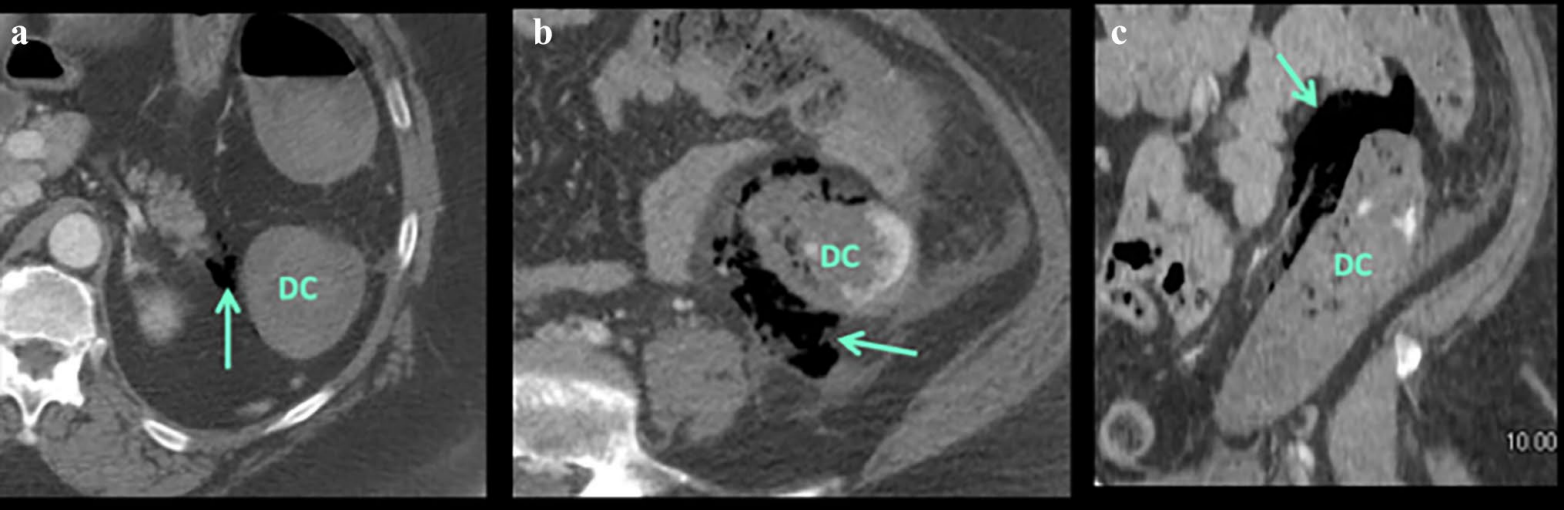
Case illustrating the left colonic compartment in a 68-year-old patient with history of breast cancer on chemotherapy presenting with abdominal pain. **a** Axial and **b** Coronal CT sections shows a small amount of extraluminal gas along the descending colon (labelled DC) in the left colonic compartment of the anterior pararenal space (teal arrows). This allowed for a preoperative diagnosis of descending colon perforation. At surgery, a perforated ulcer in the descending colon was noted secondary to chemotherapy

**Fig. 18:**
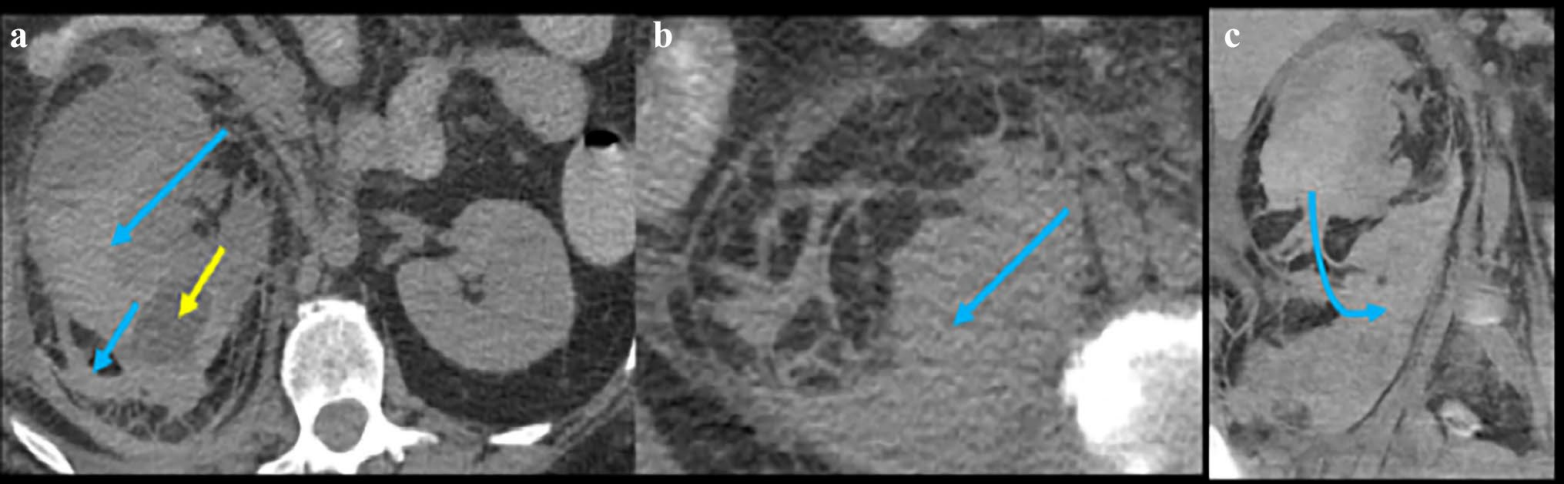
Case illustrating the perirenal space in a 44-year-old patient with intracranial extension of fungal sinusitis with urine culture showing candida and aspergillosis. Axial CT section at the **a** level of mid pole of the kidney shows an area of an area of focal hypoattenuation (yellow arrow in image a) and areas of higher attenuation (blue arrows) concerning for abscess and hemorrhage in the perirenal space. Axial CT at the **b** level of lower pole of the kidney and **c** Coronal CT section both demonstrate hemorrhage extending further inferomedially (blue arrows)

**Fig. 19:**
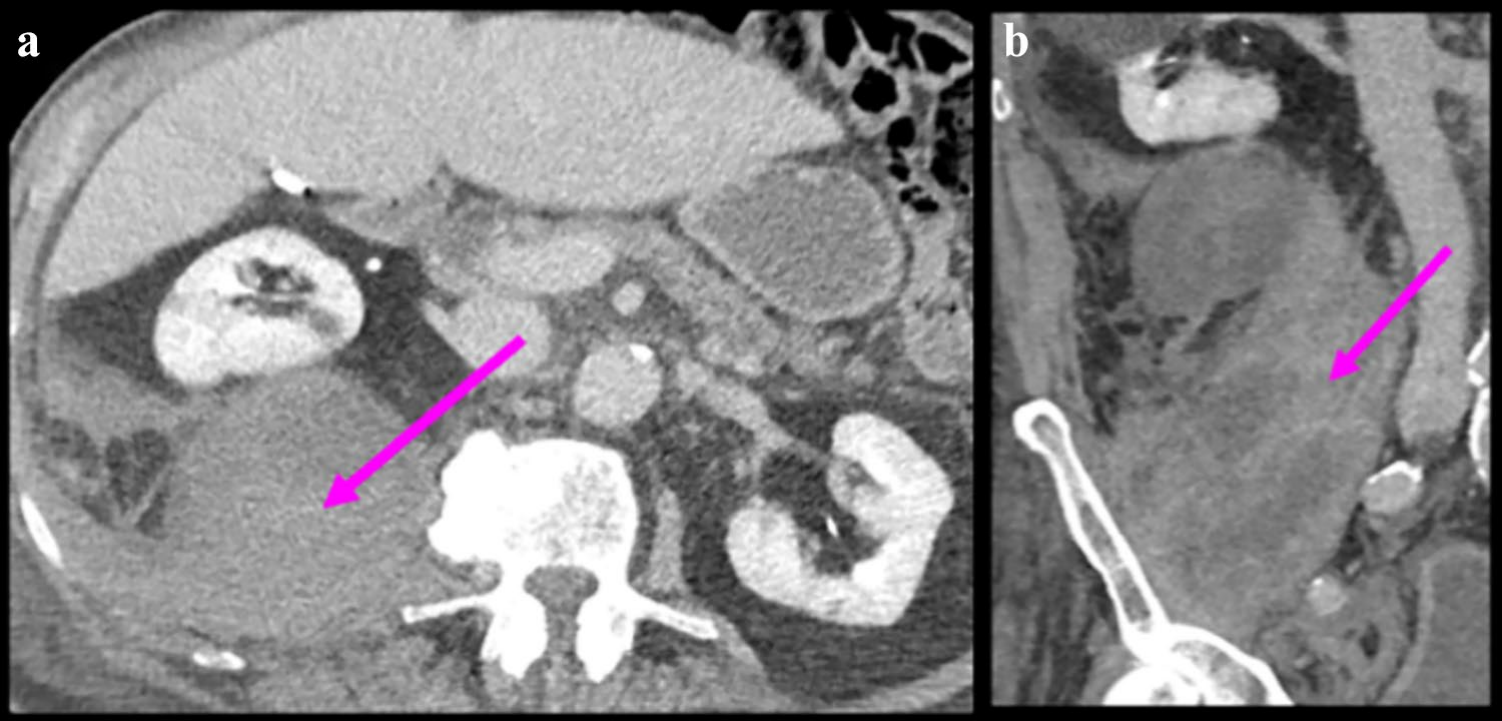
Case illustrating the posterior pararenal space in a 91-year-old patient with history of atrial fibrillation, stroke, and pulmonary embolism on therapeutic enoxaparin. **a** Axial CT and **b** coronal CT sections shows a hematoma coursing inferolaterally in the posterior pararenal space (pink arrows)

Figure 13 illustrates the spread patterns in the traditional extraperitoneal spaces. Spread of disease differs between these spaces and is useful for localization. Pathology in the anterior pararenal space localizes by its compartment. Pathology in the pancreaticoduodenal compartment localizes to the pancreas or duodenum (Figs. 14 and 15). Findings in the right colonic compartment localize to the right colon and mesocolon (Fig. 16) and findings in the left colonic compartment localize to the left colon and mesocolon (Fig. 17). Perirenal space pathology localizes to the kidneys or adrenals (Fig. 18). The posterior pararenal space does not contain organs (Fig. 19). There is a preferential pathway of spread inferolaterally in the anterior pararenal space (Figs. 14 and 15), dorsomedially to the lower pole in the perirenal space (Fig. 18) and inferolaterally in the posterior pararenal space often with superoanterior displacement of the kidney [2] (Fig. 19). Prior literature further details the extraperitoneal pelvic spaces [2, 5, 24].

## Subperitoneum

**Fig. 20:**
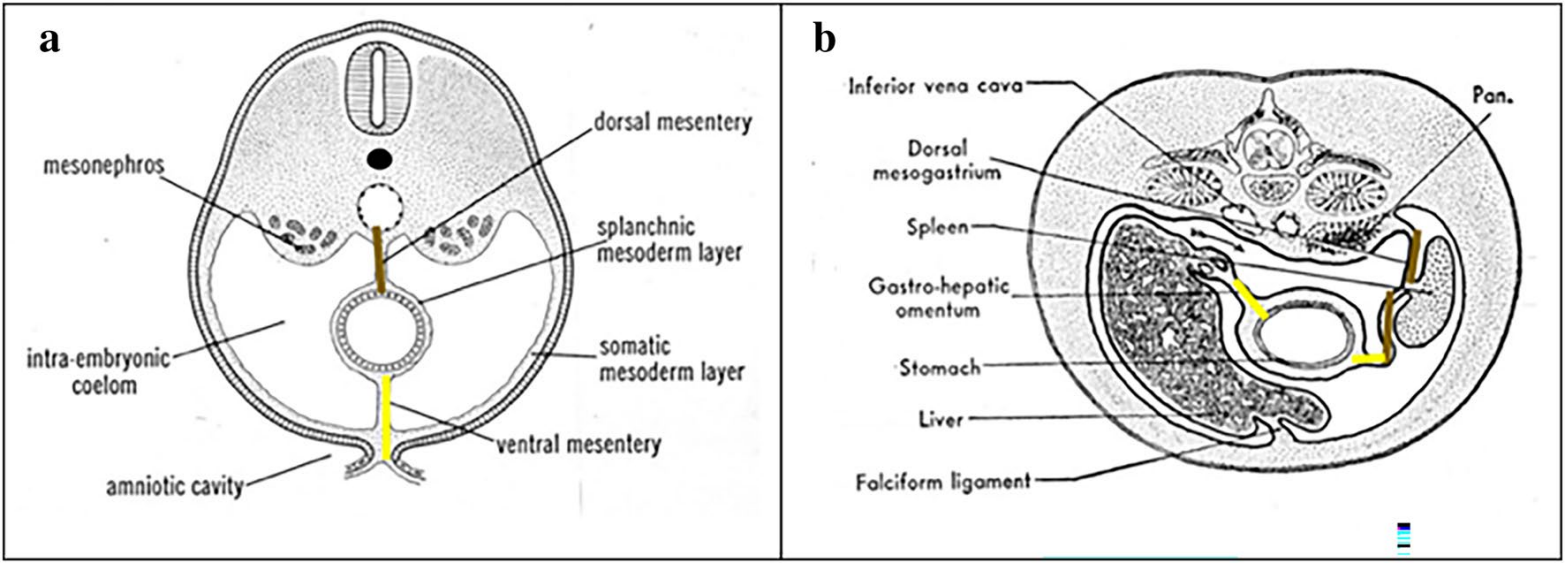
Illustration of early abdominal embryological development demonstrating the ventral (yellow line) and dorsal mesogastrium (brown line) over time spanning (**a**) to (**b**), forming interconnected pathways in the subperitoneum. Abbreviations include Pan.-pancreas. Images **a**, **b** reproduced with minor editing from “Meyers, M. A., Charnsangavej, C., Oliphant, M. (2011). *Meyers' dynamic radiology of the abdomen: normal and pathologic anatomy*. New York, NY: Springer” with permission from Springer

**Fig. 21:**
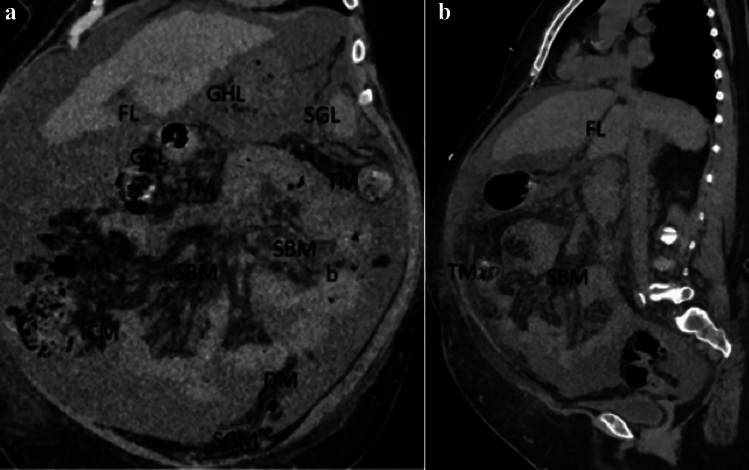
Case illustrating the difference between peritoneum and subperitoneum in a patient with diffuse ascites. Coronal (**a**) and sagittal (**b**) CT sections show diffuse ascites in the peritoneal recesses. Subperitoneal ligaments and mesentery are labeled (FL, falciform ligament; GHL, gastrohepatic ligament; GCL, gastrocolic ligament; SPL, gastrosplenic ligament; ICM, ileocolic mesocolon; AM, ascending mesocolon; TM, transverse mesocolon; DM, descending mesocolon; and SGM, sigmoid mesocolon). Note that ascites spares the mesentery, mesocolon, and ligaments in the subperitoneum

The subperitoneum is an extension of the extraperitoneum, to include the suspended abdominal organs with their associated mesenteries and ligaments [2, 5] (Figs. 20 and 21).

During embryological development, the dorsal mesogastrium (Fig. 20) gives rise to the splenorenal ligament, gastrosplenic ligament, gastrocolic ligament, and greater omentum [1–6].The ventral mesogastrium (Fig. 20) gives rise to the gastrohepatic ligament, duodenohepatic ligament, as well as hepatic ligaments such as the falciform ligament, ligamentum teres, coronary ligament, and triangular ligaments.

The dorsal mesentery extends in continuity from the dorsal mesogastrium to the duodenum, small bowel, colon, and rectum (Fig. 20). These mesenteries are named by their organ attachments: mesoduodenum, jejunal and ileal mesentery, mesocolon, and mesorectum [2]. After fusions, the suspended bowel include the first portion of the duodenum, the small bowel, the transverse colon, and the sigmoid colon. Note that the entire dorsal mesentery remains in continuity and supplies the arteries, veins, lymphatics, and nerves to their respective organs.

**Fig. 22:**
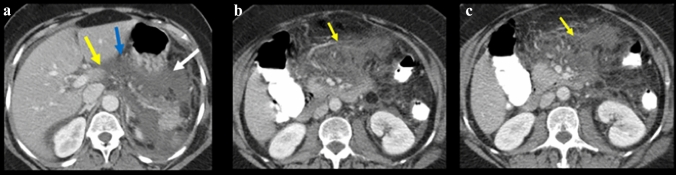
Case illustrating mesentery, ligaments, and mesocolon in a 68-year-old patient with history of pancreatitis. Axial CT section **a** demonstrates peripancreatic fluid spread to the gastrohepatic ligament (blue arrow), hepatoduoenal ligament (yellow arrow), and gastrocolic ligament (white arrow). Axial CT sections **b** and **c** shows spread of inflammation into the transverse mesocolon and small intestine mesentery (yellow arrows)

**Fig. 23:**
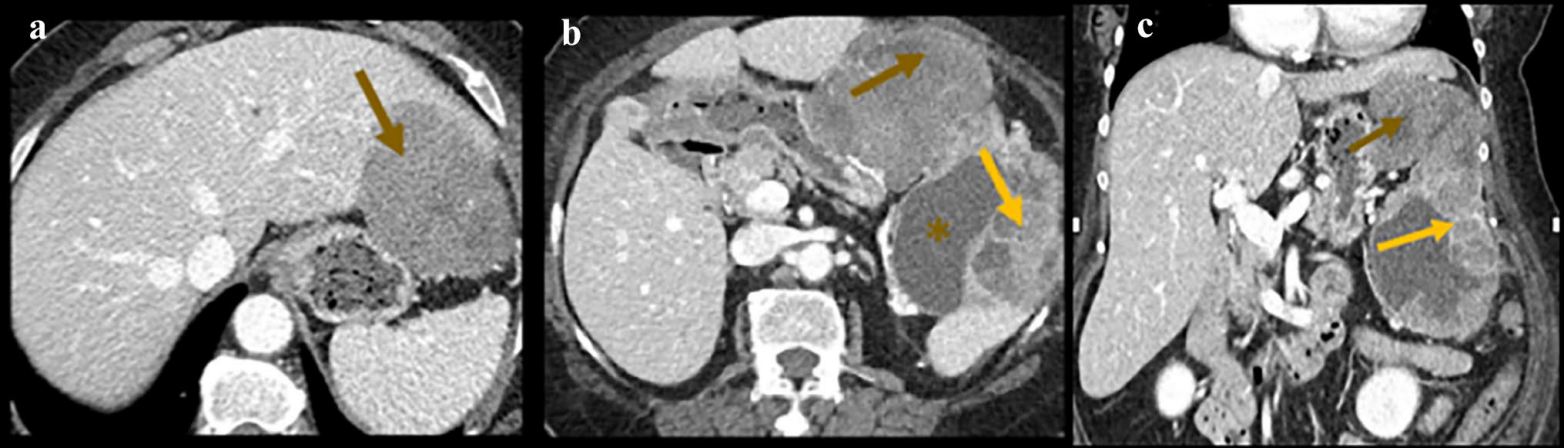
Case illustrating ligaments and mesocolon in a 62-year-old-patient who presented with pain while sleeping on her left side. Axial CT sections image **a** show a mass along the gastrocolic ligament (brown arrow), which on axial CT section **b** shows further extension into the greater omentum (brown arrow), gastrosplenic ligament (brown star), along with invasion into the spleen (orange arrow). Pathology revealed a paraganglioma. Coronal CT section **c** demonstrates these findings in a different view

**Fig. 24:**
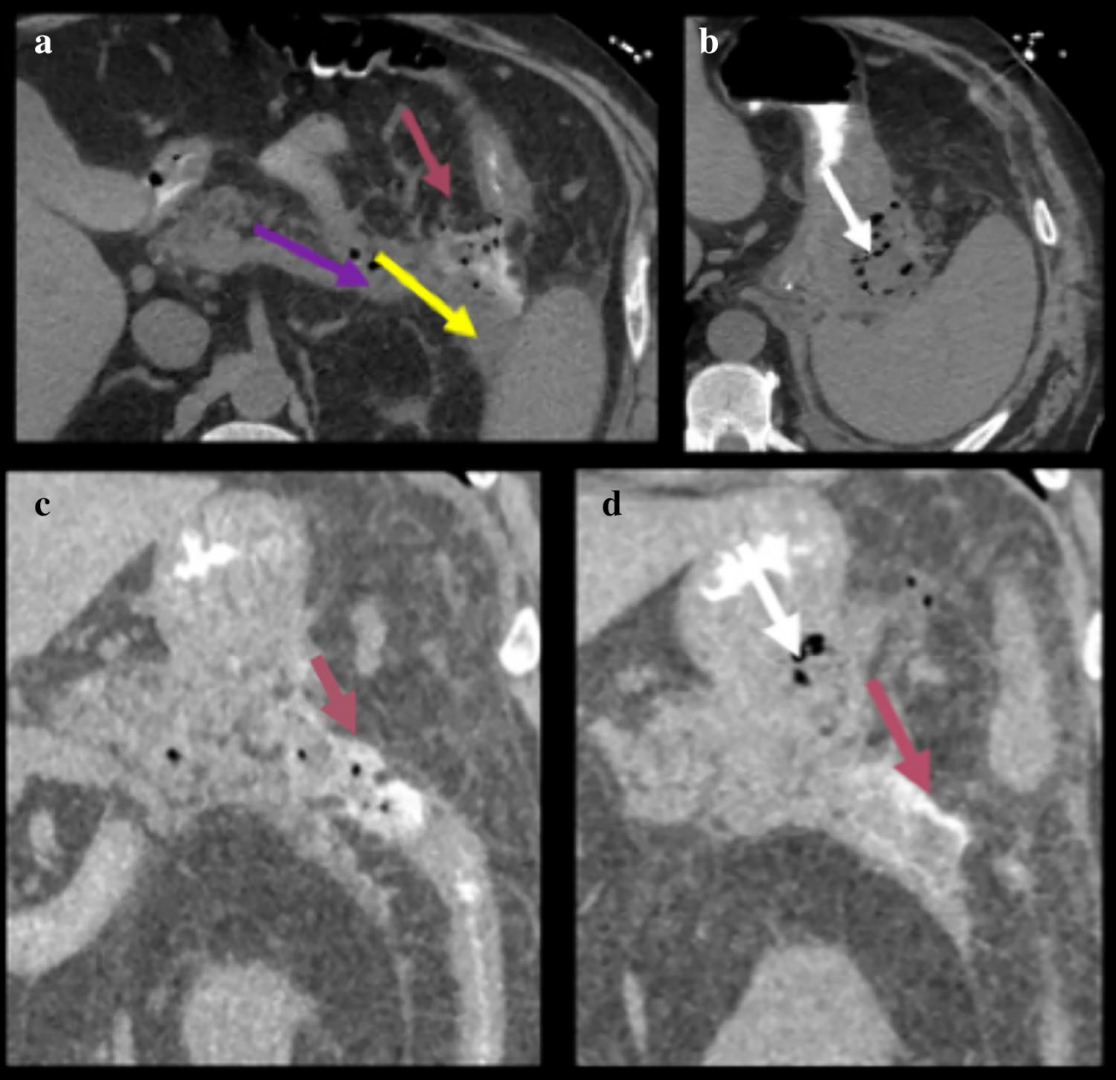
Case illustrating ligaments and mesocolon in a 77-year-old patient presenting with necrotizing pancreatitis. Axial CT sections (**a** and **b**) and coronal CT sections (**c** and **d**) demonstrates fluid and gas in the anterior pararenal space (purple arrow in image a) which tracks into the transverse colon and mesocolon with extraluminal contrast (dark magenta arrow in image **a**, **c** and **d**) compatible with a secondary transverse colonic perforation. Gas and fluid tracks along the splenorenal (yellow arrow in image **a**) and gastrosplenic (white arrow in image **b**) ligaments as well. Note how disease can spread to different organs by the interconnection of various mesenteries and ligaments

Interconnection persists between the ventral and dorsal mesogastrium and the remaining dorsal mesentery [2]. Thus, the subperitoneal space includes the extraperitoneal spaces lying deep to the parietal peritoneum, and its continuity with the suspended mesenteries and organs lying deep to the visceral peritoneum, to form one contiguous space [1–6]. The subperitoneal space contains all the vascular, lymphatics, and nerve supply of the abdominal and pelvic organs. It also provides the pathway for bidirectional spread of disease to and from any organs or mesentery within the abdomen and pelvis [1–5, 16, 25]. Examples include the complex spread of pancreatitis (Fig. 22), spread of disease between suspended organs and mesentery (Fig. 23), and extraperitoneal organs, suspended organs, and mesentery (Fig. 24).

## Conclusion

The peritoneum cavity is subdivided by the attachments of ligaments and mesenteries into interconnecting recesses. Knowing this anatomy and the flow of interperitoneal fluid explains the pathway of interperitoneal disease spread. The extraperitoneum is a space between the peritoneum and transversalis fascia, distinct from the peritoneum with a separate pathway of disease spread. This is stratified by the renal fascia into the anterior and posterior pararenal spaces, as well as the perirenal space. The anterior pararenal space is divided into the pancreaticoduodeal, left colonic, and right colonic compartments. These spaces and the connected organs have unique pathways of disease spread from each other as well compared to the peritoneal recesses. The subperitoneal space is an extension of the extraperitoneum. This includes the suspended organs along with their ligaments, mesenteries, and vessels which allows for pathways of bidirectional spread between the extraperitoneum and the suspended organs distinct from peritoneal spread of disease.
